# Differential Role for Activating FcγRIII in Neointima Formation After Arterial Injury and Diet-Induced Chronic Atherosclerosis in *Apolipoprotein E-*Deficient Mice

**DOI:** 10.3389/fphys.2020.00673

**Published:** 2020-06-17

**Authors:** Yaw Asare, Janine Koehncke, Jaco Selle, Sakine Simsekyilmaz, Joachim Jankowski, Gansuvd Shagdarsuren, Johannes E. Gessner, Jürgen Bernhagen, Erdenechimeg Shagdarsuren

**Affiliations:** ^1^Institute for Stroke and Dementia Research (ISD), LMU University Hospital, Ludwig Maximilian University of Munich (LMU), Munich, Germany; ^2^Institute for Molecular Cardiovascular Research (IMCAR), University Hospital, RWTH Aachen University, Aachen, Germany; ^3^Translational Experimental Pediatrics – Experimental Pulmonology, Department of Pediatric and Adolescent Medicine, University of Cologne, Cologne, Germany; ^4^Experimental Vascular Pathology, Cardiovascular Research Institute Maastricht (CARIM), Maastricht University, Maastricht, Netherlands; ^5^Department of Nephrology, School of Medicine, Mongolian National University of Medical Sciences, Ulaanbaatar, Mongolia; ^6^Molecular Immunology Research Unit, Clinical Department of Immunology and Rheumatology, Hannover Medical School, Hanover, Germany; ^7^Vascular Biology, Institute for Stroke and Dementia Research (ISD), LMU University Hospital, Ludwig Maximilian University of Munich (LMU), Munich, Germany; ^8^Munich Heart Alliance, Munich, Germany; ^9^Institute for Transplantation Diagnostics and Cell Therapeutics, University Hospital and Medical Faculty, Heinrich Heine University Düsseldorf, Düsseldorf, Germany

**Keywords:** Fc gamma receptors, atherosclerosis, inflammation, neointima formation, hyperlipidemia, cytokine, complement

## Abstract

Atherogenesis and arterial remodeling following mechanical injury are driven by inflammation and mononuclear cell infiltration. The binding of immune complexes (ICs) to immunoglobulin (Ig)-Fc gamma receptors (FcγRs) on most innate and adaptive immune cells induces a variety of inflammatory responses that promote atherogenesis. Here, we studied the role of FcγRIII in neointima formation after arterial injury in atherosclerosis-prone mice and compared the outcome and mechanism to that of FcγRIII in diet-induced “chronic” atherosclerosis. *Fc*γ*rIII^–/–^/Apoe^–/–^* and control *Apoe^–/–^* mice were subjected to wire-induced endothelial denudation of the carotid artery while on high-fat diet (HFD). *Fc*γ*rIII* deficiency mitigated neointimal plaque formation and lesional macrophage accumulation, and enhanced neointimal vascular smooth muscle cell (VSMC) numbers. This went along with a reduced expression of tumor necrosis factor-α (TNF-α), monocyte chemoattractant protein-1 (MCP-1/CCL2), and vascular cell adhesion molecule-1 (VCAM-1) in the neointimal lesions. Interestingly, in a chronic model of diet-induced atherosclerosis, we unraveled a dichotomic role of FcγRIII in an early versus advanced stage of the disease. While *Fc*γ*rIII* deficiency conferred atheroprotection in the early stage, it promoted atherosclerosis in advanced stages. To this end, *Fc*γ*rIII* deficiency attenuated pro-inflammatory responses in early atherosclerosis but promoted these events in advanced stages. Analysis of the mechanism(s) underlying the athero-promoting effect of *Fc*γ*rIII* deficiency in late-stage atherosclerosis revealed increased serum levels of anti-oxidized-LDL immunoglobulins IgG2c and IgG2b. This was paralleled by enhanced lesional accumulation of IgGs without affecting levels of complement-activated products C5a or C5ar1, FcγRII, and FcγRIV. Moreover, *Fc*γ*rIII*-deficient macrophages expressed more *Fc*γ*rII*, *Tnf-α*, and *Il-1β* mRNA when exposed to IgG1 or oxLDL-IgG1 ICs *in vitro*, and peripheral CD4+ and CD8+ T-cell levels were altered. Collectively, our data suggest that deficiency of activating *Fc*γ*RIII* limits neointima formation after arterial injury in atherosclerosis-prone mice as well as early stage chronic atherosclerosis, but augments late-stage atherosclerosis suggesting a dual role of FcγRIII in atherogenic inflammation.

## Introduction

As a chronic and progressive inflammatory condition of the arterial vessel wall, atherosclerosis is initiated by the recruitment of inflammatory cells and the accumulation of oxidized low density lipoproteins (oxLDL) that jointly drive the progression of atherosclerotic plaque development (Bernhagen et al., 2007; Weber and Noels, 2011; Libby et al., 2013). Previous studies suggest that oxLDL can induce autoimmune responses as evidenced by the presence of anti-oxLDL antibodies in mouse and human atherosclerotic lesions (Salonen et al., 1992; Erkkila et al., 2000). Hence, atherosclerosis has also been viewed as an autoimmune or immune complex disease (Nilsson and Hansson, 2008).

Immunoglobulin-Fc gamma receptors (FcγRs) play an important role in the clearance of immune complexes (ICs) (Nimmerjahn and Ravetch, 2008). Accumulating data also indicate the involvement of FcγRs in inflammatory diseases mediated by ICs (Meyer et al., 1998; Baudino et al., 2008). FcγRs are important cell-surface receptors on hematopoietic cells and are able to specifically bind to immunoglobulin G (IgG). This binding capacity induces a variety of biological responses like inflammatory cell activation, phagocytosis and antibody-dependent cellular cytotoxicity as well as maintenance of immunoglobulin homeostasis (Nimmerjahn and Ravetch, 2008). Four different classes of FcγRs are present in mice: FcγRI, FcγRII, FcγRIII, and FcγRIV. These receptors are classified as “activating” (FcγRI, III, and VI) and “inhibiting” (FcγRII) receptors. In spite of their differences (activating versus inhibiting), FcγRs generally play a crucial role in the clearance of IC-containing oxidized LDL. Serum LDL-IC concentrations in patients with coronary heart disease were found to be higher than those in healthy individuals (Wang et al., 2003), and the cholesterol content of circulating ICs (CICs) correlated with the presence and severity of atherosclerosis (Burut et al., 2010). Furthermore, binding of oxLDL-IC to FcγR on macrophages can activate a variety of pro-inflammatory cell responses. These include the release of inflammatory molecules such as Tnf-α and the complement component C5a, as well as the transformation of macrophages into foam cells, an important hallmark mechanism of atherosclerosis (Kiener et al., 1995).

The complement anaphylatoxin C5a is a well-described pro-inflammatory molecule, whose effect is conveyed by binding to the C5a receptors C5aR1 and C5aR2 that are expressed on immune and vascular cells (Siciliano and Rollins, 1990). Recent studies indicate that C5a may play an important role in the regulation of FcγR-dependent responses as well as in the synergistic regulation of both C5aR1 and FcγRs. IC-induced activation of FcγRIII leads to local formation of C5a, which causes further activation of C5aR1 and up-regulation of FcγRIII (Baumann et al., 2000). These intertwined processes are generally considered necessary for cell activation and inflammatory immune responses. However, the pathogenic significance of activating FcγRIII for local C5a production and its interaction with the C5a/C5aR1-axis as well as co-regulation of C5aR1/FcγR have not been explored in atherosclerosis. Although the functional role of both FcγR common chain and FcγRIII in diet-induced atherosclerotic plaque formation in hyperlipidemic mice has been amply studied (Hernandez-Vargas et al., 2006; Kelly et al., 2010; Ng et al., 2011; Zhu et al., 2014), their specific roles in different stages of atherosclerosis have not been scrutinized. Moreover, the effect of FcγRIII on accelerated atherosclerosis after arterial injury is unexplored.

Therefore, here we studied the role of FcγRIII in neointima formation after mechanical arterial injury in comparison with its role in chronic high-fat diet-induced atherosclerosis, and determined the effects of the cooperative role of FcγRIII/C5aR1 in atherosclerotic vascular inflammation.

## Materials and Methods

### Mice

C57BL/6J *Fc*γ*rIII*-deficient mice (*Fc*γ*rIII^–/–^*) were crossed with atherosclerosis-prone C57BL/6J *Apoe*-deficient mice (*Apoe^–/–^*) to generate *Fc*γ*rIII^–/–^/Apoe^–/–^*. The knockout for both genes was confirmed using genotyping PCR. Eight-week-old female *Fc*γ*rIII^–/–^/Apoe^–/–^* and corresponding control *Apoe^–/–^* mice received a high-fat diet (HFD; 21% fat, 0.15% cholesterol and 19.5% casein (Sniff, Soest, Germany) for 4 weeks to induce early atherosclerosis or for 12 and 24 weeks to study effects in late-stage atherosclerosis. Wire-induced endothelial-denudation of the carotid artery was performed in mice receiving HFD for 1 week before and 4 weeks after surgery (*n* = 10 for each group). All animal experiments were approved by local authorities (Landesamt für Natur, Umwelt und Verbraucherschutz (LANUV), Nordrhein-Westfalen, Germany) and complied with the German animal protection law.

### Tissue Preparation

Mice were euthanized using an overdose of Ketamine (500 mg/kg)/Xylazine (50 mg/kg) and perfused with sterile phosphate-buffered saline (PBS). For RNA isolation, tissue and organs were snap-frozen in liquid nitrogen and stored at −80°C. For immunohistochemical staining, tissues and organs were either fixed in 4% paraformaldehyde (PFA) and embedded in paraffin or used for cryosectioning. Aortas were placed in 4% PFA overnight, cut longitudinally and the adventitia was removed before *en face* staining.

### Determination of Atherosclerotic Lesions

The extent of atherosclerosis was assessed on aortic roots and on thoracoabdominal aortas by staining for lipid deposition with oil-red-O (ORO) staining (Sigma-Aldrich, Deisenhofen, Germany) and quantified by computerized image analysis (Diskus Software, Hilgers, Königswinter, Germany) and Leica Qwin Imaging software (Leica Ltd., Cambridge, United Kingdom). Briefly, atherosclerotic lesion areas were measured on a constant number of 5-μm transversal sections through the heart and aortic roots. For each aortic root, the average of ORO-stained areas from six sections separated by 50 μm from each other was calculated. The thoracoabdominal aorta was opened longitudinally along the ventral midline, and lesion areas in *en face* preparations were stained with ORO. The percentage of lipid deposition was calculated by dividing the stained area by the total thoracoabdominal aortic surface. For analysis of carotid arteries, 4-μm transversal serial sections from the paraffin-embedded carotid arteries were collected on glass slides. Within a standardized distance (0–360 μm) from the bifurcation, carotid artery sections (10 sections per mouse; each separated 40 μm apart) were stained using Movat’s pentachrome stain. The areas of lumen, neointima (between lumen and internal elastic lamina), media (between internal and external elastic laminae) and area within the external elastic lamina (aEEL) were measured by planimetry using Diskus Software (Hilgers). For each mouse, data from these 10 sections were averaged to represent neointima formation along this standardized distance.

### Histological and Immunohistological Analysis

Serial sections of the aortic roots and carotid arteries were analyzed for their cellular composition by quantitative immunohistochemistry. Immunofluorescence staining was performed using antibodies against CD3 (MCA1477, AbD Serotec, Cologne, Germany), CD45R/B220 (553085, BD Bioscience, Heidelberg, Germany), myeloperoxidase (MPO, RB-373-A, Neomarkers, Freemont, CA, United States), C5aR1 (CD88, ab59390, Abcam, Cambridge, United Kingdom), α-SMA (M0851, Dako, Hamburg, Germany), Mac-2 (CL8942AP, CEDARLANE, Burlington, ON, Canada), VCAM-1, MCP-1/CCL2, and TNF-α (all from Santa Cruz, Santa Cruz, CA, United States), CD19 (clone 6D5, MCA1439, BioRad) and FITC- or Cy3-conjugated secondary antibody (Jackson ImmunoResearch, Ely, United Kingdom). Nuclei were co-stained with 4′, 6-diamidino-2-phenylindol (DAPI). Proliferating cells were determined using the Ki-67 marker (M7249, DakoCytomation) and apoptotic cells using TUNEL-staining Kit (Roche). Immunoglobulins were detected using anti-IgG (AI-9200, Vector Laboratories, Burlingame, CA, United States) and anti-IgM (sc-2075, Santa Cruz) antibodies and visualized by avidin-biotin-complex method. Lipid deposits were stained with Nile Red and Oil-red-O (Sigma-Aldrich). Collagen contents were analyzed using Gomori Trichrome staining. Images were recorded with a Leica DMLB fluorescence microscope and a charge-coupled device camera. Analysis of images was performed using Diskus analysis software (Hilgers) with the exception of Nile Red stainings, which were analyzed using Image J software (National Institute of Health).

### RNA Extraction and cDNA Synthesis

Aorta tissues were homogenized in RLT buffer (Qiagen, Hilde, Germany) using Homogenator (Qiagen) and stainless-steel beads (74104, Qiagen). Macrophages were disrupted in RLT buffer + 1% β-mercapto-ethanol. Total RNA was purified using RNeasy spin column kit (Qiagen) and the concentration was determined by measuring the absorbance at 260 nm using a Nanodrop spectrophotometer (GE Healthcare, Freiburg, Germany). Equal concentrations (1 μg) were transcribed into cDNA using the High Capacity cDNA Reverse Transcription Kit (Applied Biosystems, Heidelberg, Germany) according to manufacturer’s protocols.

### Real-Time PCR

Gene expression was determined by real-time PCR with primers for mouse *Tnf-α, Il-1β, Tgf-β, Il-6, Il-10, Il-4* (Sigma) and *C5ar1, Fcgr3a, Fcgr2b* (Biorad, Munich, Germany) in a thermal cycler 7900HT (Applied Biosystems). The expression of target genes was calculated by the ΔΔCt method and normalized to GAPDH. All RT-PCR reactions were carried out in duplicate. A preparation without template served as a negative control.

### ELISA

Plasma was obtained from blood that was centrifuged at 3000 *g* for 20 min. Immunoglobulin titers against oxidized low-density lipoprotein (oxLDL) and malondialdehyde-oxidized (MDA-LDL) in plasma were measured using sandwich ELISA. 96-well ELISA plates (Nunc, Dreieich, Germany) were coated overnight at 4°C with 10 μg/μl oxidized LDL or MDA-LDL. After washing with PBST, plasma dilutions of 1:100 were added and incubated for 2 h at RT. Detection occurred for 1 h at RT with mouse anti-IgG (sc-2005), anti-IgM (sc-2064), anti-IgA (sc-3791), anti-IgG2a (sc-2061), anti-IgG2b (sc-2062), and anti-IgG3 (sc-2063) antibodies (Santa Cruz) labeled with horseradish peroxidase (HRP). Substrate was added and chemiluminescence was measured at 450 nm using an ELISA reader (Tecan, Männedorf, Switzerland). The C5a plasma titer was determined using a C5a detection Kit (ELM-ccC5a-001, Raybiotech, Peachtree Corners, GA, United States). Total Plasma cholesterol and triglyceride levels were quantified using enzymatic assays (Analyticon, 4046 and 5052, Lichtenfels, Germany) according to the manufacturer’s protocol. Plasma levels of IL-10 (88-7105-8), IL-6 (88-7064-88) and Tnf-α (88-7324-88, Bioscience, Würzburg, Germany) were determined by using ELISA kits.

### Flow Cytometry

Cells were isolated from spleen and lymph nodes and a single-cell suspension was prepared and filtered over MACS pre-separation filter (Miltenyi, Bergisch Gladbach, Germany). Thereafter, cells were treated with erythrocyte lysis buffer (0.155 M NH_4_Cl, 10 mM NaHCO_3_). Cell suspensions were carefully washed and stained with FACS staining buffer and combinations of antibodies against T and B cells: FITC-anti-CD3, APC-anti-CD4, PE-Cy7-anti-CD8, PE-anti-CD25, Per-CP-Cy5.5-anti-CD19 and APC-Cy7-anti-CD45. For regulatory T cells: PerCP-Cy5.5-anti-CD3, FITC-anti-CD4, PE-anti-CD25, APC-Cy7-anti-CD45 and APC-anti-Foxp3. All antibodies were obtained from eBioscience (Vienna, Austria) and were incubated for 60 min on ice. Mouse regulatory T-cell staining kit (eBioscience) was used for permeabilization and fixation of cells for intracellular staining of Foxp3. At least 100.000 gated cells were acquired, after appropriate fluorescence compensation, and analyzed in a FACSCanto II using FACSDiva software (BD Biosciences). Final analysis was performed using FlowJo software (Tree Star Inc.).

### OxLDL and Immune Complex Preparation

To prepare oxLDL, human plasma LDL (437644, Calbiochem, Heidelberg, Germany) was diluted with sterile PBS to a final concentration of 1.5 mg/ml and incubated with copper sulfate (5 μM) at 37°C for 4 h (mild oxidation) or overnight (heavy oxidation). The reaction was stopped using 25 μl EDTA (5 mM), and oxidized LDL (oxLDL) was purified using PD10 desalting columns (GE healthcare). Final elution was done in 3.5 ml sterile PBS and oxLDL preparations stored at 4°C in the dark. The concentration was determined using Bradford assay. Malondialdehyde (MDA)-LDL from human plasma was purchased from Hölzel (Cologne, Germany). ICs were produced by incubating oxLDL (30 μg/ml) and mouse anti-oxLDL-IgG1 (OB40, University of Graz; 100 μg/ml) in sterile PBS at 4°C overnight. The concentration was determined using Lowry assay.

### Isolation and Stimulation of Macrophages

Bone marrow-derived macrophages (BMDMs) were isolated from femur and tibia of *Apoe^–/–^* and *Fc*γ*rIII^–/–^*/*Apoe^–/–^* mice as already established (Asare et al., 2017). Briefly, bones were isolated, cut open with a sterile scissor and flushed with ice-cold sterile PBS using a syringe with a 27-G needle. The bone marrow was filtered through a 40 μm cell strainer (Greiner) and collected in a 50 ml falcon. The cells were then centrifuged at 1200 rpm for 5 min, resuspended in culture medium (RPMI 1640 + L-Glucose, 10 mM HEPES, 10% FCS, 100 U/ml Gentamycin, 15% LCM) and plated in 15 cm bacterial plastic plates (Greiner). After 7 days of differentiation, the macrophages were used for stimulation experiments. The cells were transferred into 6-well plates (Greiner, Frickenhausen, Germany), cultured overnight without LCM and synchronized for 2 h without FCS. The stimulation of macrophages was done by adding 10 μg/ml and 50 μg/ml oxidized LDL, IgG or ICs for 6 h at 37°C; unstimulated macrophages served as control. RNA was isolated, reverse transcribed and used for RT-PCR.

### Statistical Analysis

All statistical analysis was performed using GraphPad Prism 5 (GraphPad software Inc.). All data are given as means ± SEM and were analyzed for normality by the Kolmogorov–Smirnov test or D’Agostino and Pearson omnibus test and then by the 2-tailed Student *t* test or two-way ANOVA with Bonferroni post-test as appropriate. *P* < 0.05 was considered statistically significant.

## Results

### *Fc*γ*rIII* Deficiency Limits Neointima Expansion in Atherosclerosis-Prone Mice

**FIGURE 1:**
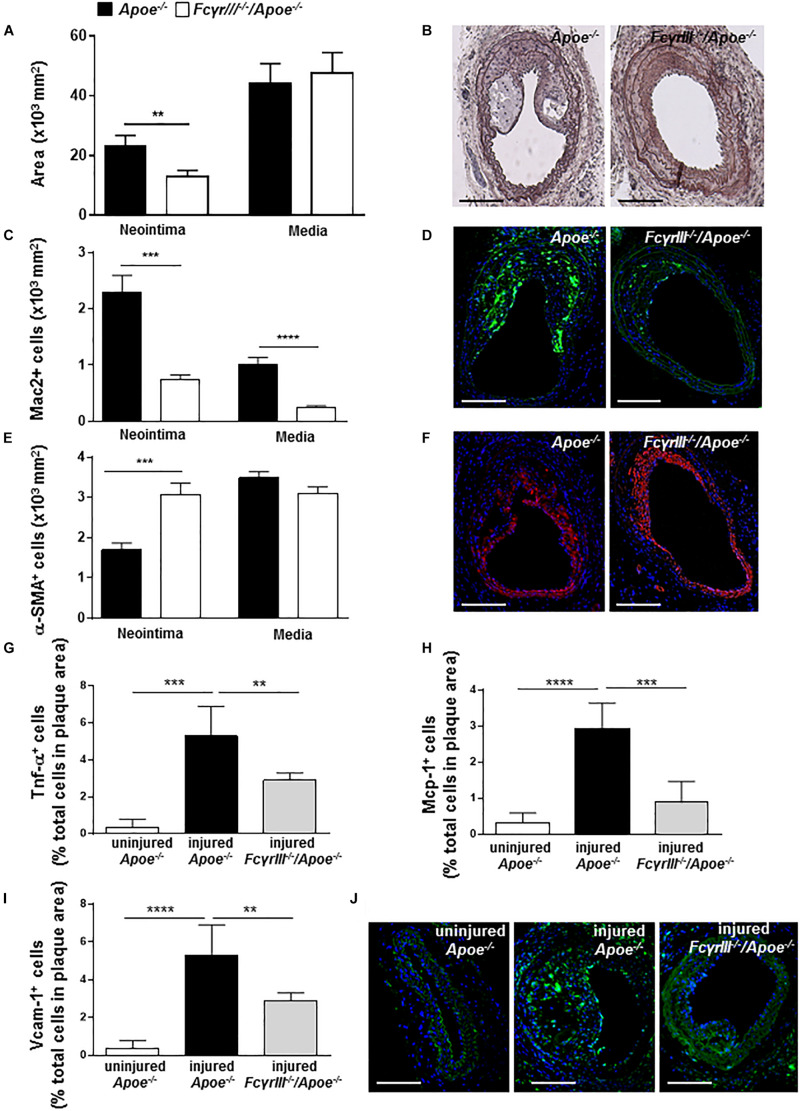
*Fc*γ*rIII* deficiency limits neointima expansion in atherosclerosis-prone mice. **(A)** Quantification of neointimal and medial area in *Fc*γ*rIII^–/–^/Apoe^–/–^* and *Apoe^–/–^* mice 4 weeks after wire-induced injury in carotid arteries. **(B)** Representative images of Movat’s pentachrome staining. Scale bars 200 μm. **(C)** Quantification of neointimal and medial Mac2^+^ macrophages and **(D)** representative immunofluorescence images for Mac-2 (green). **(E)** Quantification of neointimal and medial α-SMA^+^ smooth muscle cells and **(F)** representative α-SMA staining (red). Scale bars 200 μm. Cell nuclei are in blue. **(G)** Quantification of neointimal Tnf-α^+^, **(H)** Mcp-1^+^, and **(I)** Vcam-1^+^ cells. **(J)** Representative immunofluorescence images for Vcam-1 (green) are shown. Graphs represent the mean ± SEM (*n* = 6–7 mice per group). 2-tailed *t*-test, *Fc*γ*RIII^–/–^/Apoe^–/–^* vs. *Apoe^–/–^* mice. **p < 0.01, ***p < 0.001, ****p < 0.0001.

To study the effect of *Fc*γ*rIII* deficiency in neointima formation after arterial injury, *Fc*γ*rIII^–/–^/Apoe^–/–^* and control *Apoe^–/–^* mice received an HFD 1 week before and 4 weeks after endothelial denudation of the left common carotid artery. *Fc*γ*rIII* deficiency attenuated neointimal plaque formation when compared to control mice, whereas the medial area was unaltered (Figures 1A,B). Analysis of the cellular composition in the neointimal plaques and medial area revealed significantly reduced Mac-2^+^ macrophages (Figures 1C,D and Supplementary Figure S1A) and an increase in α-SMA^+^ vascular smooth muscle cell (VSMC) numbers whereas medial VSMC numbers were not affected (Figures 1E,F and Supplementary Figure S1B) in *Fc*γ*rIII-*deficient mice. Also, the neointimal collagen content and IgG area did not differ between control *Apoe^–/–^* and *Fc*γ*rIII-*deficient mice (Supplementary Figures S1C,D). Furthermore, injury-induced Tnf-α^+^ cells were reduced in *Fc*γ*rIII-*deficient neointimal plaques (Figure 1G) and this went along with reduced expression levels of downstream adhesion molecules and chemokines. Specifically, we found significantly lower numbers of cells expressing Mcp-1/Ccl2 and Vcam-1 upon *Fc*γ*rIII* deficiency compared to control mice (Figures 1H–J), consistent with the limited expansion of the neointima observed in *Fc*γ*rIII-*deficient mice. Collectively, these data indicate that FcγRIII promotes pro-inflammatory responses in the vasculature to accelerate neointima formation after arterial injury.

### Dichotomic Role of FcγRIII in Chronic Atherosclerosis

We next compared the observed effect of FcγRIII in neointimal lesion formation with that in a model of early atherosclerosis, i.e., *Fc*γ*rIII^–/–^/Apoe^–/–^* versus *Apoe^–/–^* mice on a 4-week HFD in the absence of mechanic injury. In line with previous reports (Zhu et al., 2014), *Fc*γ*rIII* deficiency conferred atheroprotection in early stages of the disease and limited the expression of inflammatory cytokines and adhesion molecules (Supplementary Figures S2A–E).

**FIGURE 2:**
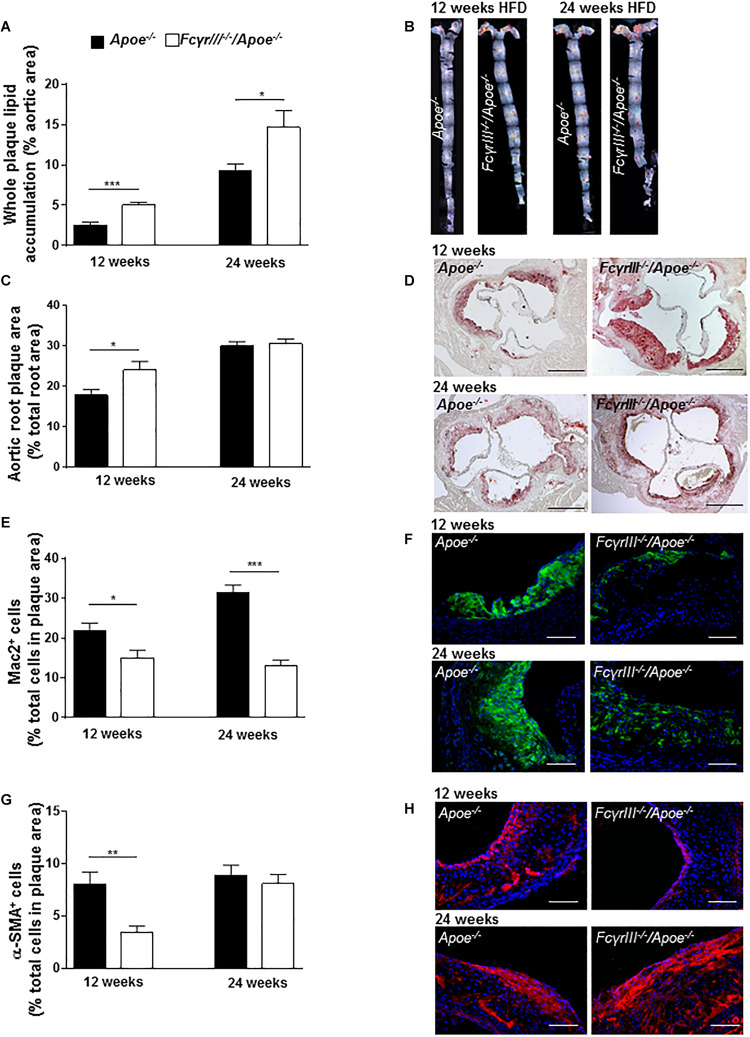
Dichotomic role of FcγRIII in chronic atherosclerosis. **(A–H)** *Fc*γ*rIII^–/–^ Apoe^–/–^*, and *Apoe^–/–^* mice received HFD for 12 and 24 weeks. **(A)** Quantification of lesion size in whole aorta (*n* = 6–9 mice per group). **(B)** Representative image of en face stained whole aorta lesions. **(C)** Quantification of lesion sizes in aortic root (*n* = 10–12 mice per group). **(D)** Representative image of Oil-Red-O stained aortic root lesion. Scale bar 500 μm. **(E)** Quantification of Mac-2 + macrophages (green). **(F)** Representative Mac-2 immunostaining (green). **(G)** Quantification of α-SMA smooth muscle cells. **(H)** Representative α-SMA immunostaining (red). Magnification × 40; scale bar 100 μm; and (*n* = 10 mice per group). Graphs represent mean ± SEM. 2-tailed *t*-test, *p < 0.05, **p < 0.01, ***p < 0.001.

We also studied the role of FcγRIII in intermediate and late-stage atherosclerosis in atherogenic mice receiving an HFD for 12 and 24 weeks, respectively. The analysis revealed an unexpected and previously unrecognized phenotype, in which *Fc*γ*rIII* deficiency promoted atherosclerosis in the aorta at both 12 and 24 weeks of HFD as determined by *en face* staining (Figures 2A,B). *Fc*γ*rIII* deficiency also led to an atheropromoting effect in aortic root in mice receiving a 12-week HFD regimen, while no effect was seen in aortic root after 24 weeks of HFD (Figures 2C,D). To further explore this unexpected finding, serial sections of the aortic roots were analyzed by quantitative immunohistochemistry to determine the cellular plaque composition. Lesional Mac-2^+^ macrophages were significantly decreased in *Fc*γ*rIII^–/–^/Apoe^–/–^* compared to control *Apoe^–/–^* mice after both 12 and 24 weeks of HFD (Figures 2E,F), but this went along with an increased necrotic core size (Supplementary Figure S3). Similarly, α-SMA+ VSMC content was significantly reduced after 12 but not 24 weeks of HFD (Figures 2G,H). Lesional total T lymphocyte (CD3^+^) and MPO^+^ neutrophil content as well as Ki-67^+^ proliferative and TUNEL^+^ apoptotic cells did not differ between both groups (Supplementary Table S1) and neither did the expression of C5a, *C5ar1*, *Fc*γ*RII*, and *Fc*γ*RIV* (Supplementary Figures S4A–F). However, while there are technical limitations regarding the reliable quantification of lesional T-cell subsets, *Fc*γ*rIII* deficiency led to altered splenic and lymph node CD4+, CD8+, and FoxP3+ T-cell subset levels as measured by flow cytometry (Supplementary Figures S5A–D). Together, these data indicate that *Fc*γ*rIII* deficiency has a dichotomic role in chronic atherosclerosis of hyperlipidemic atherogenic mice, attenuating early lesion formation but augmenting intermediate-late-stage atherosclerosis.

### *Fc*γ*rIII* Deficiency Enhances the Accumulation of Immunoglobulins in Intermediate-to-Advanced Atherosclerotic Lesions

**FIGURE 3:**
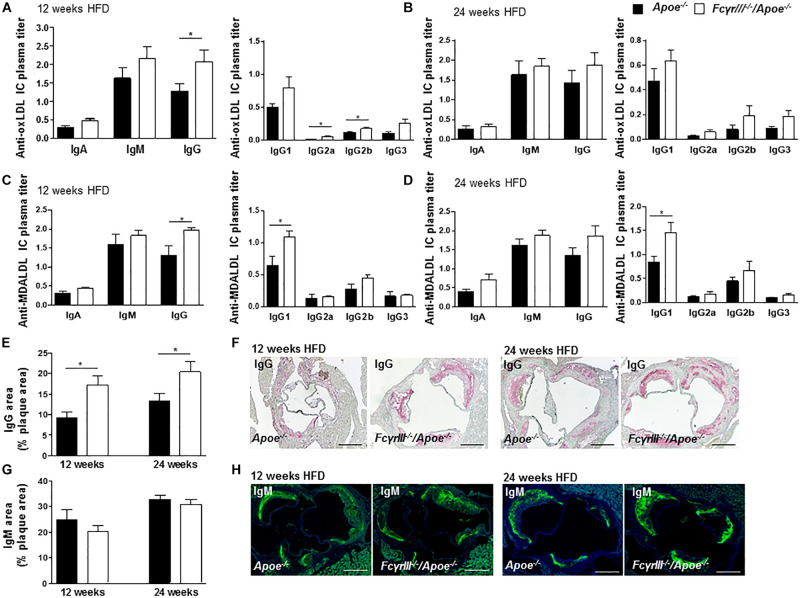
*Fc*γ*rIII* deficiency enhances the accumulation of immunoglobulins in intermediate-to-advanced atherosclerotic lesions. **(A–H)** *Fc*γ*rIII^–/–^Apoe^–/–^*, and *Apoe^–/–^* mice received HFD for 12- and 24-weeks. **(A,B)** Quantification of anti-oxLDL IgA, IgM, IgG, and IgG subtypes IgG2a, IgG2b, and IgG3 in plasma from mice fed HFD for 12 weeks **(A)** and for 24 weeks **(B)**. **(C,D)** Quantification of plasma levels of anti-MDA-LDL in mice fed HFD for 12 weeks **(C)** and for 24 weeks **(D)**. **(E)** Quantification of accumulated IgG antibodies in aortic root lesions. **(F)** Representative IgG staining. **(G)** Quantification of accumulated IgM antibodies in aortic root lesions. **(H)** Representative IgM staining. Scale bar 500 μm. Graphs represent mean ± SEM (*n* = 6). 2-tailed *t*-test, *p < 0.05.

Fc gamma receptor aggregation mediates inflammatory responses induced by autoantibodies and ICs (Ben Mkaddem et al., 2019) and the progression of atherosclerosis is characterized by the presence of disease-promoting autoantibodies against modified LDL (Shoenfeld et al., 2004). To investigate whether anti-oxidized low-density lipoprotein (oxLDL) and anti-malondialdehyde-oxidized (MDA-LDL) antibodies may explain the observed increased plaque size in *Fc*γ*rIII^–/–^/Apoe^–/–^* mice in intermediate-late-stage atherosclerosis, we measured serum levels of anti-oxLDL and anti-MDA-LDL IgM, IgA, and IgG antibody subtypes. At 12 weeks of HFD, the levels of circulating IgG, IgG2a, and IgG2b anti-oxLDL antibodies were significantly increased in *Fc*γ*rIII^–/–^/Apoe^–/–^* mice compared to control mice (Figure 3A), whereas only a trend for increased IgG (subtype) levels was noted after 24 weeks of HFD (Figure 3B). IgA and IgM titers showed no significant differences between both groups at both intervals of HFD. Analysis of anti-MDA-LDL antibodies revealed significantly enhanced IgG and IgG1 titer in *Fc*γ*RIII^–/–^/Apoe^–/–^* mice after 12 weeks of HFD compared to *Apoe^–/–^* mice (Figure 3C). After 24 weeks of HFD, the anti-MDA-LDL IgG1 titer was increased in *Fc*γ*rIII^–/–^/Apoe^–/–^* mice compared to *Apoe^–/–^* mice. Differences for all other anti-MDA-LDL Ig subtypes did not reach significance at either time interval (Figure 3D). To more directly examine the functional consequences of elevated circulating Ig antibody subtypes following *Fc*γ*rIII* deficiency on atherosclerotic lesion formation, serial sections of aortic roots were analyzed by quantitative immunohistochemistry for an accumulation of IgG and IgM whole fractions. Our analysis revealed significantly increased IgG concentrations in the lesions of *Fc*γ*rIII^–/–^/Apoe^–/–^* mice at both time points of HFD (Figures 3E,F), whereas IgM content did not differ between groups (Figures 3G,H). Given that plaque IgG-type antibodies are generally considered to be atherogenic, while IgMs predominantly exhibit atheroprotective activity (Schmitz et al., 2018; Sage et al., 2019), these findings suggest the need of FcγRIII for clearance of pro-atherogenic ICs against modified lipoproteins, and may at least partly explain the exacerbated atherosclerotic lesion formation observed upon *Fc*γ*rIII* deficiency.

### *Fc*γ*rIII* Deficiency Induces Pro-inflammatory Profile in Macrophages

**FIGURE 4:**
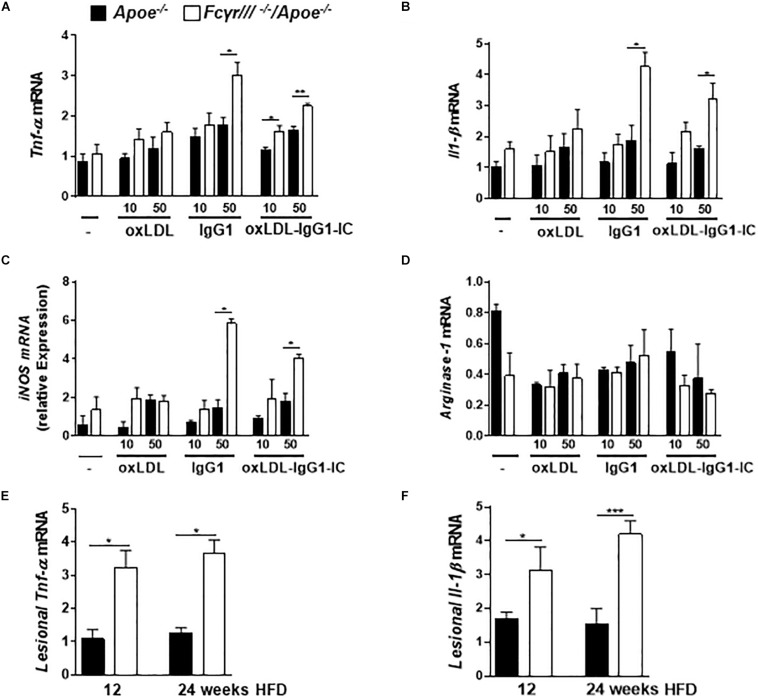
*Fc*γ*rIII* deficiency induces pro-inflammatory profile in macrophages. **(A–D)** BMDMs from *Fc*γ*rIII^–/–^/Apoe^–/–^*, and *Apoe ^–/–^* mice were stimulated with varying concentrations of oxidized LDL, IgG1 and oxLDL-IgG1 immune complexes (IC) for 6 h. Quantification of *Tnf-*α **(A)**, *Il-1*β **(B)**, *iNOS* **(C)**, and Arginase-1 **(D)** mRNA levels. *n* = 3 independent experiments. **(E,F)** *Fc*γ*RIII^–/–^/Apoe^–/–^*, and *Apoe ^–/–^* mice received HFD for 12- and 24- weeks. Quantification of lesional *Tnf-*α **(E)** and *Il-1*β **(F)** in aortic tissues. *n* = 6 mice per group. Graphs represent the mean ± SEM. two-tailed *t*-test, *Fc*γ*rIII^–/–^/Apoe^–/–^* vs. *Apoe^–/–^* mice, *p < 0.05, **p < 0.01, ***p < 0.001.

Based on the reported role of FcγRIII in regulating the decision between pro- and anti-inflammatory responses by inducing ITAM or inhibitory ITAM (ITAMi) signaling (Aloulou et al., 2012), we determined the phenotype of macrophages in *Fc*γ*rIII^–/–^/Apoe^–/–^* mice. BMDMs were stimulated with two different concentrations of oxidized LDL, anti-oxLDL-IgG1, and soluble ICs for 6 h. Analysis of gene expression revealed an increase of *Tnf-*α, *Il-1*β, *iNOS, and Fc*γ*rII* upon *Fc*γ*rIII* deficiency, while the expression of *Arginase-1*, *Tgf-*β, *Il-10*, and *C5ar1* did not differ in both groups (Figures 4A–D and Supplementary Figures S6A–D). Of note, quantification of pro-inflammatory gene expression in the atherosclerotic lesions showed increased lesional *Tnf-*α and *Il-1*β levels in *Fc*γ*rIII*-deficient mice (Figures 4E,F). Collectively, these findings indicate that *Fc*γ*rIII* deficiency enhances pro-inflammatory responses in the bone marrow-derived compartment of the vasculature to promote advanced atherosclerosis.

## Discussion

Our study investigated the role of activating FcγRIII in neointima expansion after arterial injury and in diet-induced chronic atherosclerosis in *Apolipoprotein E*-deficient hypercholesterolemic mice, comparing the phenotype in early versus intermediate-to-late stages. Using a wire injury-based accelerated atherosclerosis model, we demonstrate that *Fc*γ*rIII* deficiency reduces neointimal plaque size and macrophage content and induces a stable plaque phenotype with an increased VSMC compartment, which was paralleled by reductions in proinflammatory cytokine TNF-α, the chemokine MCP-1/CCL2, and the atherogenic adhesion molecule VCAM-1 in the vasculature. The lesion-lowering protective effect of *Fc*γ*rIII* deficiency was mirrored in a model of diet-induced “early” atherogenesis. In contrast, in a diet-induced model of “advanced” atherosclerosis, *Fc*γ*rIII* deficiency exacerbated atherosclerotic plaque formation after 12 and 24 weeks of HFD. This was accompanied by lesional accumulation of IgG-type immunoglobulins and elevated serum autoantibodies against modified-LDL and lipid levels. Of note, these unexpected atheropromoting effects of *Fc*γ*rIII* deficiency were independent of the cooperativity between the C5a/C5aR1 and FcγRIII/FcγRII axis; hence supporting their independent role in the pathogenesis of a different disease context.

Neointima formation after endothelial denudation is driven by intima exposure, platelet adhesion, activation of inflammatory genes, and increased leukocyte trafficking into the injured vessel wall. To restore the integrity of the artery, phenotypically changed VSMCs accumulate in the intimal layer to maintain vascular stability. Likewise, the extent of macrophage infiltration is a strong determinant of neointimal lesion size (Schober and Weber, 2005). Hence, inhibiting genes that regulate leukocyte trafficking including Mac-1, VCAM-1, and MCP-1/CCL2 has generally resulted in reduced neointimal mass, indicating a decisive role of inflammation in neointimal formation (Shah, 2003). Our data indicating a protective role of *Fc*γ*rIII* deficiency in neointima formation after arterial injury is attributable to the reduction of lesional inflammatory cells and VCAM-1, MCP-1/CCL2, and TNF-α levels.

Our findings that *Fc*γ*rIII* deficiency exacerbates intermediate-to-late-stage atherosclerosis at first sight contradicts previously reported data, according to which *Fc*γ*rIII* deficiency attenuated atherosclerotic plaque formation (Kelly et al., 2010; Zhu et al., 2014). In *Fc*γ*rIII^–/–^/Ldlr^–/–^* mice, Kelly et al. (2010) observed fewer lesions after 24 weeks of HFD. While it is difficult to reconcile these findings, it is worth noting that *Apoe^–/–^* mice on HFD develop extensive late-stage atherosclerosis with different characteristics than the corresponding *Ldlr^–/–^* mice receiving HFD for the same duration (Veniant et al., 2001). Notwithstanding their limitations and differences, both models have been instrumental in identifying specific pathways that can be targeted for atheroprotection in humans (Gleissner, 2016). Using 5-week-old *Fc*γ*rIII^–/–^/Apoe^–/–^* mice fed an HFD for 10 weeks, Zhu et al. (2014) reported a reduced lesion size accompanied by a decreased foam cell content. These conditions are closer to the conditions used in our 12-week HFD model of intermediate stage atherosclerosis, but differences still apply. In fact, HFD in our intermediate model of *Fc*γ*rIII^–/–^/Apoe^–/–^* mice was not started until the age of 8 weeks and the applied diet in our model was 2 weeks longer. The model applied by Zhu et al. (2014) may thus be closer to our 4-week HFD model of early atherosclerosis. Indeed, in this model, we found *Fc*γ*rIII* deficiency to confer atheroprotection in 8-week-old mice. In conjunction, the data by Kelly, Zhu and colleagues and the data in our current study indicate a Janus-faced role of FcγRIII in atherosclerosis, exhibiting a pro-atherogenic role in early atherosclerosis and/or younger mice, whereas this effect appears to be overcompensated by a FcγRIII-mediated athero-protective mechanisms in more advanced stages of the disease. Whether the alterations in splenic and lymph node T cells that we observed in our study upon *Fc*γ*rIII* deficiency contribute to this effect is unclear and clearly needs future scrutiny. The decrease in FoxP3+ Tregs in the 12-week HFD model could support this notion, but Tregs were found to be elevated in *Fc*γ*rIII^–/–^/Apoe^–/–^* mice at 24 weeks of HFD, and the analysis of CD4+ and CD8+ subsets provided a complex picture as well.

Autoantibodies are produced against oxidized LDL, but whether the titer of anti-oxLDL autoantibodies serves as a marker for atherosclerosis progression is still a unclear (Shoenfeld et al., 2004). Anti-oxLDL IgGs are known to be pro-atherogenic by forming IC with oxLDL (Mallavia et al., 2013), which bind to FcγR and lead to a pro-inflammatory cell response by macrophage activation. Anti-oxLDL IgM antibodies have been suggested to play a protective role in atherosclerosis (Faria-Neto et al., 2006) and ICs block the oxLDL uptake by macrophages. We found that the IgM anti-oxLDL response remained unchanged in plasma of *Fc*γ*rIII*-deficient mice, whereas the IgG anti-oxLDL antibodies were increased. Further analysis revealed an increase in the IgG2a/b response accompanied by a slight increase in IgG1, suggesting a pro-inflammatory effect of ICs in lesion progression. Additionally, clinical studies have shown that IgG antibodies directed against another form of modified LDL, MDA-LDL, correlate with cardiovascular diseases (Salonen et al., 1992). We observed an increased IgG and IgG1 response in plasma upon *Fc*γ*rIII* deficiency, indicating a role for anti-MDA-LDL antibodies in the progression of lesion formation. Furthermore, autoantibodies against oxLDL have been detected in atherosclerotic lesions (Shoenfeld et al., 2004) and plaques are known to contain large amounts of IgM and IgG (Yla-Herttuala et al., 1994). This could be a result of diffusion as well as deposition of Igs and ICs from the circulation into the intima (Burut et al., 2010). In our current study, we found an increased accumulation of IgG in *Fc*γ*RIII-*deficient lesions, while IgM deposition did not differ between both groups. Hence, indicating that deficiency of *Fc*γ*rIII* is accompanied by a defective clearance of anti-oxLDL ICs leading to lesional accumulation of IgG.

Our study also offers a mechanistic explanation for the observed dichotomy. Monomeric IgG1 and oxLDL-IgG1 IC-triggered cytokine production is enhanced in *Fc*γ*rIII*-deficient macrophages, insinuating an anti-inflammatory role for FcγRIII, at least in this experimental paradigm. Likewise, in a mouse model of obstructed kidney, an anti-inflammatory effect of FcγRIII was seen in IVIg (intravenous immunoglobulin) and 2.4G2 F(ab)2 (anti-FcγRIII/II antibody)-treated mice (Aloulou et al., 2012). On the other hand, a decrease in TNF-α was found after MDA-LDL-IC incubation of CD16 siRNA-transfected macrophages (Zhu et al., 2014) and bone marrow-transplanted *Fcr*γ*-chain*-deficient mice (Mallavia et al., 2013). ICs used in these studies consisted of all types of IgG, whereas we used mouse IgG1 which, similar to IVIg, was shown to control the inflammatory response by ITAMi (Aloulou et al., 2012). In addition, FcγRIII is the only activating receptor that is able to bind to IgG1 and small ICs can mimic the response mediated by IVIg (Siragam et al., 2005). Although previous studies have proven that even at high concentrations of IgG1, FcγRIII is the mediating receptor in *Fc*γ*rII*-deficient models (Siragam et al., 2006; Aloulou et al., 2012), we cannot completely exclude possible effects from FcγRII or paired FcγRII/FcγRIII complexes. Moreover, dual activating and inhibitory effects of FcγRIII, depending on the degree of aggregation of its natural ligand IgG, have been described (Pinheiro da Silva et al., 2007).

In summary, we present evidence for a protective effect of *Fc*γ*rIII* deficiency in neointima expansion and in early atherosclerosis. This is attributable to reduced pro-inflammatory responses in the vasculature. However, in advanced atherosclerosis *Fc*γ*rIII* deficiency augments lesion formation, at least in part through enhanced accumulation of Igs in the atherosclerotic lesions. It will be intriguing to identify the triggers of this Ig-mediated response in the future. Gaining a better insight into *Fc*γ*RIII* function in atherosclerosis will provide valuable information for the rational design of anti-atherosclerotic therapeutics.

## Data Availability Statement

The raw data supporting the conclusions of this article will be made available by the authors, without undue reservation, to any qualified researcher. Requests to access the datasets should be directed to yaw.asare@med.uni-muenchen.de.

## Ethics Statement

The animal study was reviewed and approved by the Landesamt für Natur, Umwelt und Verbraucherschutz (LANUV), Nordrhein-Westfalen, Germany.

## Author Contributions

JK, YA, JS, and SS performed the experiments and analyzed the data. ES and YA planned and supervised the experiments. ES, YA, and JB wrote the manuscript. YA, ES, JB, JJ, GS, and JG edited and revised the manuscript. All authors contributed to the article and approved the submitted version.

## Conflict of Interest

The authors declare that the research was conducted in the absence of any commercial or financial relationships that could be construed as a potential conflict of interest.
