# Supplementary material for: Differential Role for Activating FcγRIII in Neointima Formation After Arterial Injury and Diet-Induced Chronic Atherosclerosis in Apolipoprotein E-Deficient Mice
Source: Front Physiol. 2020 Jun 17;11:673. doi: 10.3389/fphys.2020.00673 (PMC7313534; doi:10.3389/fphys.2020.00673)

SUPPLEMENTAL FIGURES WITH LEGENDS

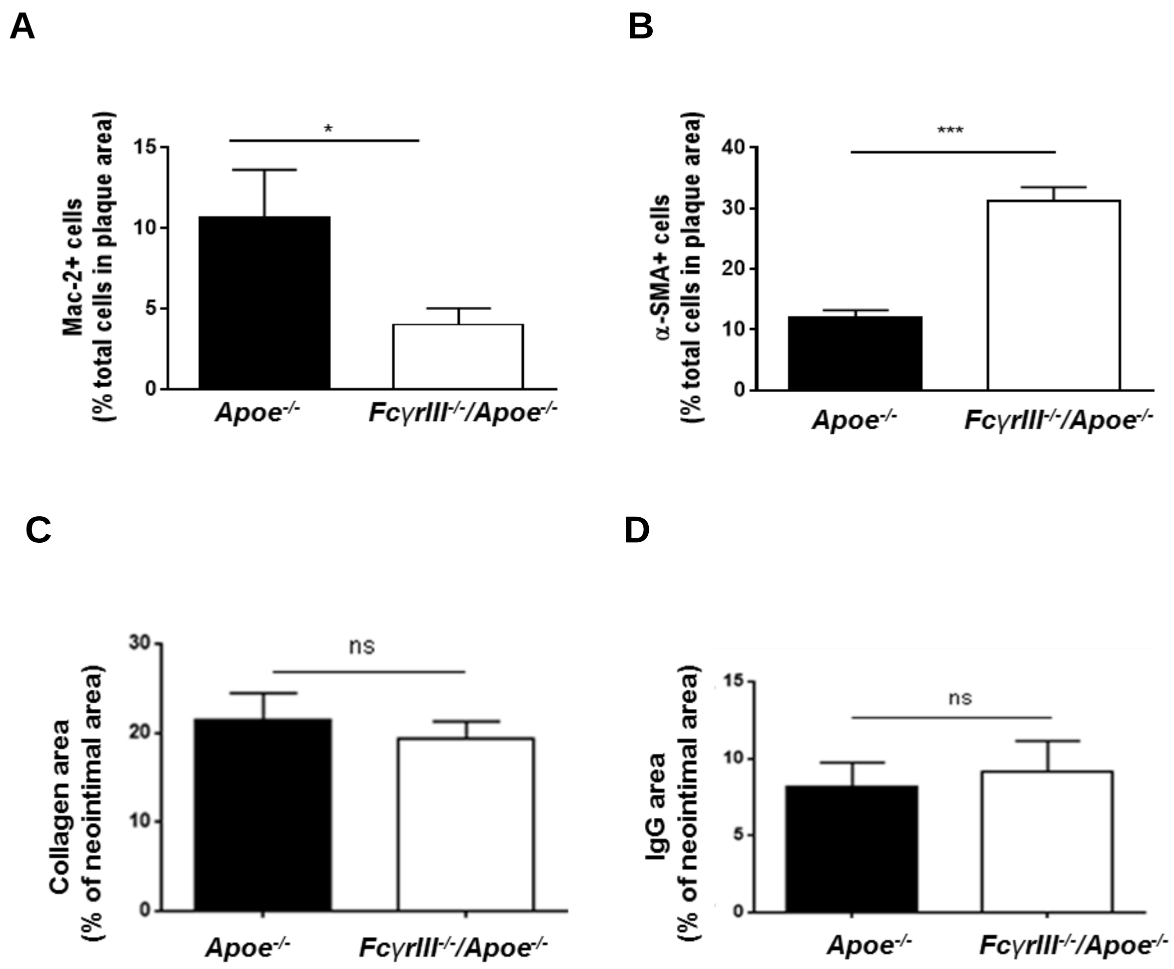

Supplementary Figure S1. Effect of *FcγrIII* deficiency on neointimal plaque composition. Quantification of Mac2+ cells (A), α-SMA+ cells (B), collagen content (C), and IgG accumulation (D) in *FcγrIII*$^{-/-}$/*Apoe*$^{-/-}$ and *Apoe*$^{-/-}$ mice 4 weeks after wire-induced injury in carotid arteries. Graphs represent the mean ± SEM (n=6-7 mice / group). Two-tailed t-test, *FcγrIII*$^{-/-}$/*Apoe*$^{-/-}$ vs. *Apoe*$^{-/-}$ mice. *p< 0.05, **p< 0.01, ***p<0.001. ns indicates not significant.

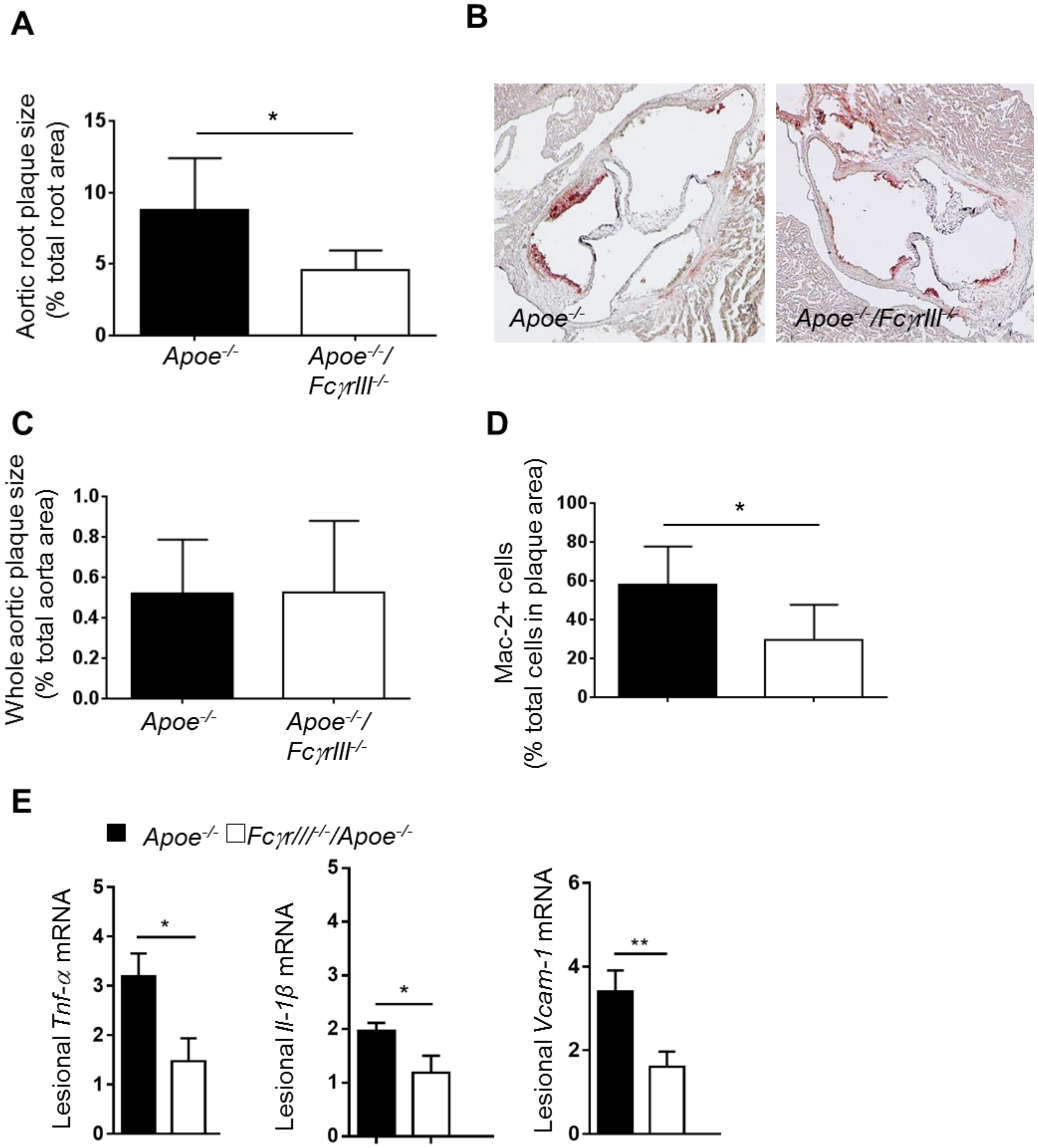

Supplementary Figure S2. *FcγrIII* deficiency reduces early lesion formation in hyperlipidemic mice. (**A** - **E**) *FcγrIII-/-Apoe-/-* and *Apoe-/-* mice received HFD for 4 weeks. (**A**) Quantification of aortic root lesion sizes. (**B**) Representative Oil-Red-O stained lesion. (**C**) Quantification of lesions in whole aorta. (**D**) Quantification of lesional macrophage content. (**E**) Quantification of *Tnf-α*, *Il-1β*, and *Vcam-1* mRNA in lesions. Graphs represent the mean ± SEM (n=6-7 mice per group). Two-tailed t-test, *FcγrIII*$^{-/-}$/*Apoe*$^{-/-}$ vs. *Apoe* $^{-/-}$ mice. *p< 0.05.

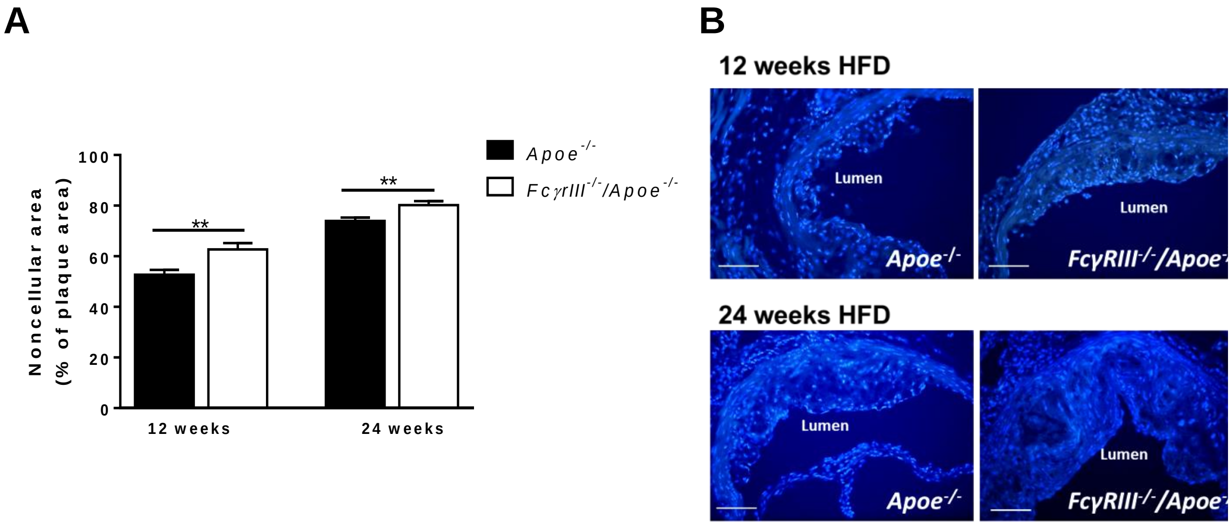

Supplementary Figure S3. *FcγrIII* deficiency induces necrotic core expansion. (**A**) Quantification of noncellular areas in *FcγrIII-/-Apoe-/-* and *Apoe-/-* mice fed HFD for 12- and 24- weeks. Graphs represent the mean ± SEM (n=10-12 mice per group). (**B)** Representative images of DAPI-staining. Magnification x40; scale bars 100µm; Two-tailed t-test, *FcγrIII*$^{-/-}$*/Apoe*$^{-/-}$ vs. *Apoe* $^{-/-}$ mice. *p< 0.05.

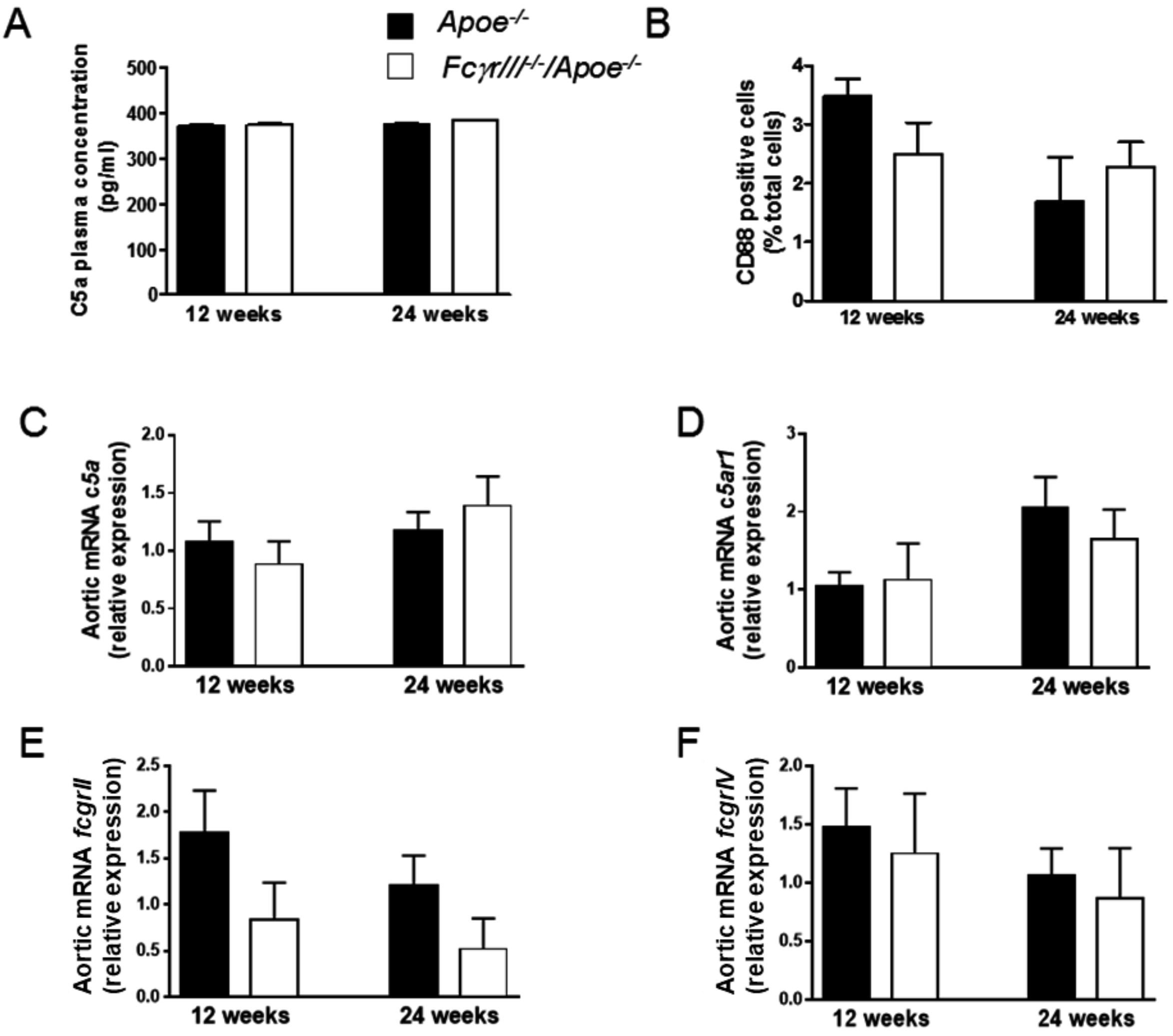

Supplementary Figure S4. Effect of *FcγrIII* deficiency on the expression of C5a, *C5ar1*, *FcγrII* and *FcγrIV in vivo.* (**A**-**F**) *FcγrIII-/-Apoe-/-* and *Apoe-/-* mice received HFD for 12 and 24 weeks. (**A**) Measurement of complement component C5a plasma levels (n=6). (**B**) Quantification of C5ar1-positive cells ($CD88^{+}$) in aortic root lesion (n=6). Quantification of mRNA expression levels of (**C**) *C5a*, (**D**) *C5ar1*, (**E**) *FcγrII,* and (**F**) *FcγrIV* in whole aorta as determined by RT-PCR (n=6). Graphs represent the mean ± SEM. Results of 3 repeated measurements.

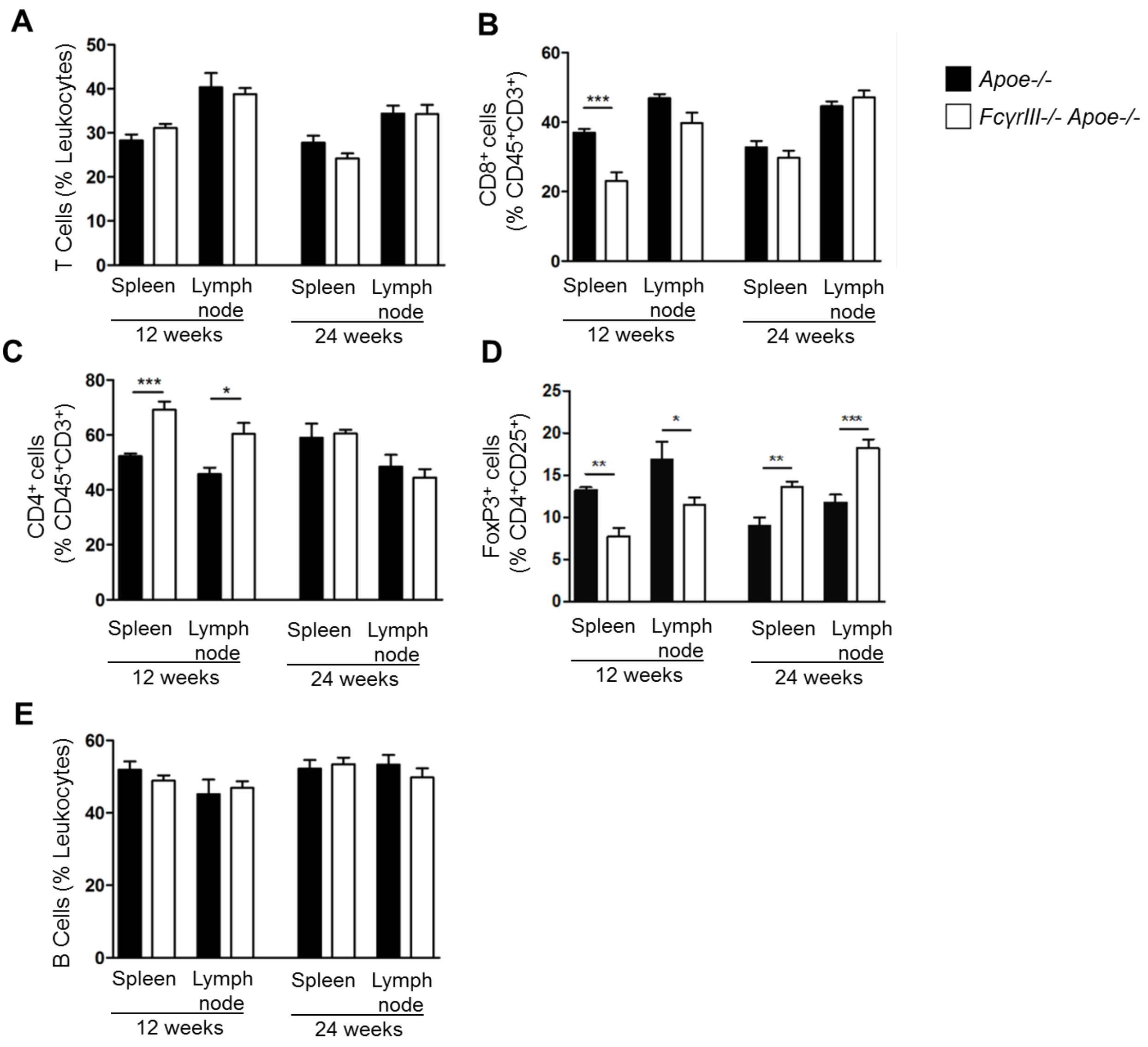

Supplementary Figure S5. Effect of *FcγrIII* deficiency on leukocyte subsets. *FcγrIII*$^{-/-}$ *Apoe-/-* and *Apoe-/-* mice received HFD for 12 and 24 weeks. Thereafter spleen and lymph nodes were harvested for analysis by flow cytometry. Quantification of CD3^{+} T cells (**A**), CD8^{+} cells (**B**), CD4^{+} cells (**C**), CD4^{+}CD25^{+}FoxP3^{+} cells (**D**), and CD19^{+} B cells (**E**). Data are mean ± SEM (n=6-9). Two-tailed t-test, *FcγrIII*$^{-/-}$/*Apoe*$^{-/-}$ vs. *Apoe* $^{-/-}$ mice. *p< 0.05; **p< 0.01; ***p< 0.001.

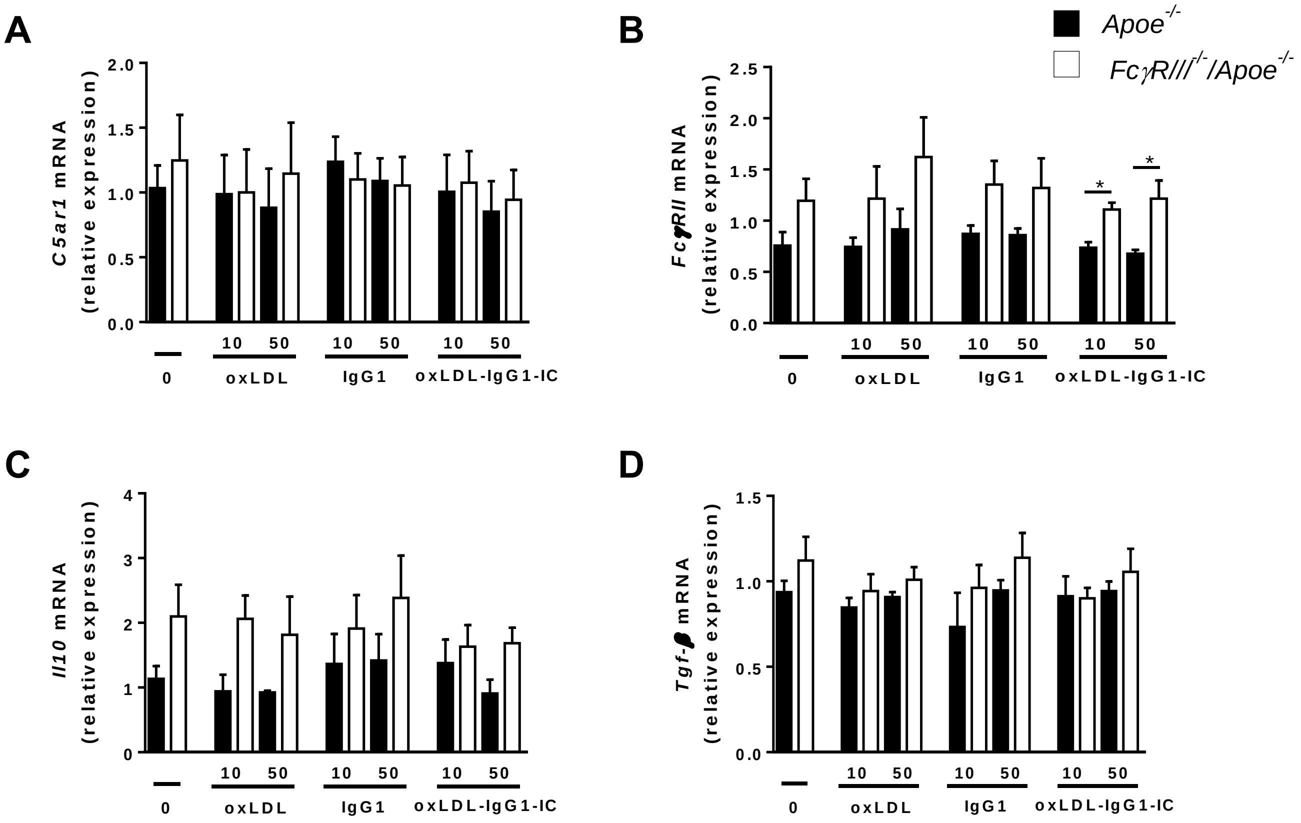

Supplementary Figure S6. Effect of *FcγrIII* deficiency pro-inflammatory responses in macrophages. (**A-D**) Bone marrow-derived macrophages (BMDMs) from *FcγrIII*$^{-/-}$*/Apoe*$^{-/-}$ and *Apoe*$^{-/-}$ mice were isolated and stimulated with 10 µg/ml or 50 µg/ml oxLDL, IgG1 and oxLDL-IgG1 immune complexes (IC) for 6 h. Quantification of mRNA for *C5ar1* (**A**), *FcγrII* (**B**), *Il-10* (**C**), and *Tgf-β* (**D**) by RT-PCR. Graphs represent the mean ± SEM (n=3 repeated experiments). Two-tailed t-test, *FcγrIII*$^{-/-}$*/Apoe*$^{-/-}$ vs. *Apoe* $^{-/-}$ mice. *p< 0.05.

Supplementary Table S1 and associated representative images. Quantification of plaque cellular composition. T lymphocyte (CD3$^+$), B Lymphocyte (CD19$^+$), neutrophils (MPO$^+$), proliferating (Ki67$^+$) and apoptotic (TUNEL$^+$) cells were stained in aortic root lesions of *FcγrIII*$^{-/-}$/*Apoe*$^{-/-}$ and *Apoe*$^{-/-}$ mice. Numbers represent the mean ± SEM (n=10).

	12 weeks HFD		24 weeks HFD	
	% of total cells in plaque area		% of total cells in plaque area	
	Apoe$^{-/-}$	*FcγrIII*$^{-/-}$/*Apoe*$^{-/-}$	*Apoe*$^{-/-}$	*FcγrIII*$^{-/-}$/*Apoe*$^{-/-}$
**CD3$^+$**	0.241 ± 0.160	0.277 ± 0.180	0.172 ± 0.115	0.448 ± 0.159
**CD19$^+$**	0.065 ± 0.07	0.007 ± 0.126	0.1363 ± 0.190	0.210 ± 0.190
**MPO$^+$**	0.475 ± 0.322	0.390 ± 0. 251	0. 265 ± 0.104	0.343 ± 0.290
**Ki67$^+$**	0 ± 0	0 ± 0	0.174 ± 0.132	0.0334 ± 0.066
**TUNEL$^+$**	0.454 ± 0.392	0.311 ± 0.175	0.0613 ± 0.119	0.0722 ± 0.056

Shown below are representative images. CD3$^+$ T lymphocytes (green), CD19$^+$ B lymphocytes (green), MPO$^+$ neutrophils (red), Ki67$^+$ proliferating (green) and TUNEL$^+$ apoptotic cells (red) were stained in aortic root lesions of *FcγrIII*$^{-/-}$*Apoe*$^{-/-}$ and *Apoe*$^{-/-}$ mice fed HFD for 24 weeks. Cell nuclei staining with DAPI (blue). White arrows indicate positive stained cells. Scale bars 100 μm.

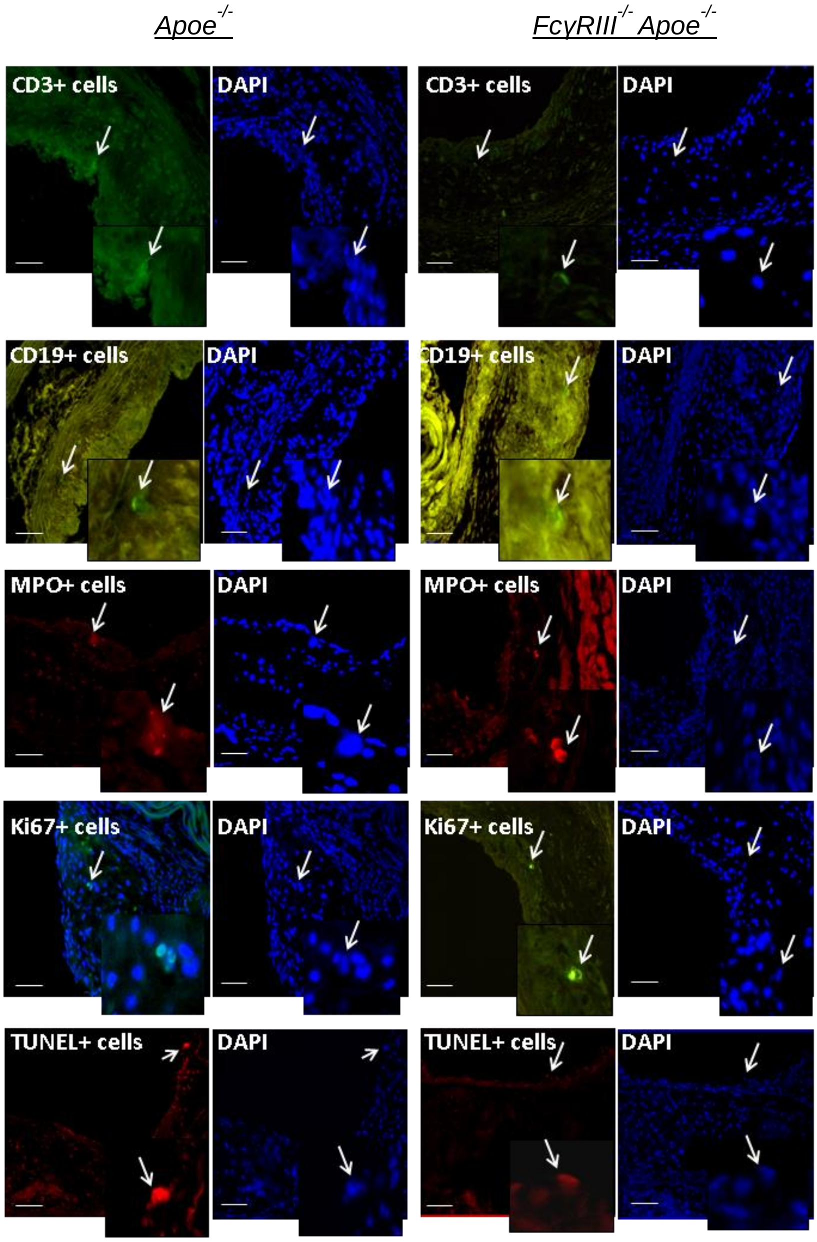

Supplement: Supplementary file 1 [file Data_Sheet_1.pdf]
